# A Versatile Peroxidase from the Fungus *Bjerkandera adusta* Confers Abiotic Stress Tolerance in Transgenic Tobacco Plants

**DOI:** 10.3390/plants10050859

**Published:** 2021-04-23

**Authors:** Nancy Sofia Hernández-Bueno, Ramón Suárez-Rodríguez, Edgar Balcázar-López, Jorge Luis Folch-Mallol, José Augusto Ramírez-Trujillo, Gabriel Iturriaga

**Affiliations:** 1 Centro de Investigación en Biotecnología, Universidad Autónoma del Estado de Morelos, Av. Universidad 1001, Cuernavaca 62209, Morelos, Mexico; nancy.hernandez@uaem.mx (N.S.H.-B.); rsuarez@uaem.mx (R.S.-R.); jordi@uaem.mx (J.L.F.-M.); augusto.ramirez@uaem.mx (J.A.R.-T.); 2 Departamento de Farmacobiología, Centro Universitario de Ciencias Exactas e Ingeniería, Universidad de Guadalajara, Blvd. M. García Barragán # 1451, Guadalajara 44430, Jalisco C.P., Mexico; edgar.balcazar@academicos.udg.mx; 3 División de Estudios de Posgrado e Investigación, Tecnológico Nacional de México/I.T. Roque, Km. 8 Carretera Celaya-Juventino Rosas, Roque, Celaya 38110, Guanajuato, Mexico

**Keywords:** abiotic stress, *Bjerkandera adusta*, transgenic tobacco, versatile peroxidase

## Abstract

White-rot fungi are efficient lignin degraders due to the secretion of lignin peroxidase, manganese peroxidase, laccase, and versatile peroxidase (VP) on decayed wood. The VP is a high-redox-potential enzyme and could be used to detoxify reactive oxygen species (ROS), which accumulate in plants during biotic and abiotic stresses. We cloned the *VP* gene and expressed it via the *Agrobacterium* transformation procedure in transgenic tobacco plants to assay their tolerance to different abiotic stress conditions. Thirty independent T_2_ transgenic VP lines overexpressing the fungal *Bjerkandera adusta* *VP* gene were selected on kanamycin. The VP22, VP24, and VP27 lines showed significant manganese peroxidase (MnP) activity. The highest was VP22, which showed 10.87-fold more manganese peroxidase activity than the wild-type plants and led to a 34% increase in plant height and 28% more biomass. The VP22, VP24, and VP27 lines showed enhanced tolerance to drought, 200 mM NaCl, and 400 mM sorbitol. Also, these transgenics displayed significant tolerance to methyl viologen, an active oxygen-generating compound. The present data indicate that overproducing the *VP* gene in plants increases significantly their biomass and the abiotic stress tolerance. The VP enzyme is an effective biotechnological tool to protect organisms against ROS. In transgenic tobacco plants, it improves drought, salt, and oxidative stress tolerance. Thus, the *VP* gene represents a great potential for obtaining stress-tolerant crops.

## 1. Introduction

Reactive oxygen species (ROS) control different signaling pathways in plants involved in stress and pathogen responses, photosynthesis, programmed cell death, hormonal action growth, and development [1,2]. ROS might cause cellular injury by reacting with biological compounds, and their cell damage is one of the major mechanisms underlying the biotic and abiotic stresses including drought, high light, wounding, salt, or pathogen infection [3]. Cells have evolved highly regulated mechanisms to maintain a balance between ROS production and breakdown [4]. Peroxidase (EC 1.11.1.7) oxidizes a vast array of compounds (hydrogen donors) in the presence of H_2_O_2_. Heme-containing peroxidases form a superfamily of enzymes responsible for numerous biosynthetic and degradative functions. On the basis of their sequences and catalytic properties, non-animal heme peroxidases can be divided into three classes: I-bacterial peroxidases [5], II-fungal peroxidases [6], and III-classical plant peroxidases [7]. The most important ligninolytic enzymes of white-rot fungi, which efficiently degrade lignin and a wide range of aromatic xenobiotics, including polychlorinated phenols, nitro- and amino-substituted phenols, synthetic dyes, and polycyclic aromatic hydrocarbons [8,9,10], are phenol oxidase laccase (Lac, E.C. 1.10.3.2) and three heme peroxidases: lignin peroxidase (LiP, EC 1.11.1.14), which catalyze the oxidative cleavage of carbon–carbon bonds and ether bonds (C–O–C) in non-phenolic aromatic substrates of high redox potential [11]; the manganese peroxidase (MnP, EC 1.11.1.13), which requires Mn^2+^ to complete its catalytic cycle and forms Mn^3+^ -chelates, acting as diffusing oxidizers [12]; and versatile peroxidases (VP, EC 1.11.1.16) that has both previous activities and is present in *Pleurotus* and *Bjerkandera* fungal species, and in some other fungi such as *Lepista irina* and *Panus tigrinus* [13]. These enzymes can directly attack lignin, cellulose, and hemicellulose in the plant cell wall to decompose it. Litter-decomposing fungi of families such as Strophariaceae, Tricholomataceae, and Bolbitiaceae have been found to have an evident expression of MnP.

Another group of peroxidases has been identified in fungi and bacteria that are capable of degrading lignin, known as dye-decolorizing peroxidases (DyPs, EC 1.11.1.19) [14]. The ability of VP to oxidize both Mn^2+^ and aromatic compounds was first reported in *Pleurotus eryngii*. To the present day, most VP studies have been performed with enzymes of the white-rot fungi *Bjerkandera* sp. strain BOS55, *Bjerkandera* sp. (B33/3), *B. adusta* UAMH 8258, *Bjerkandera fumosa*, *Pleurotus ostreatus*, *P. eryngii*, and *Pleurotus pulmonarius* [15]. 

The VP catalytic mechanism is similar to classic peroxidases, in which substrate oxidation is carried out by a two-electron multistep reaction at the expense of hydrogen peroxide [16]. VP oxidize Mn^2+^ (as MnP), degrade the lignin model dimer veratryl glycerol-guaiacylether to yield veratraldehyde, and oxidize veratryl alcohol and *p*-dimethoxybenzene to veratraldehyde and *p*-benzoquinone, respectively (as reported for LiP) [10]. Their optimal pH for oxidation of Mn^2+^ to Mn^3+^ (pH 5) and aromatic compounds and dyes (pH 3) to Mn^3+^ differ and are similar to those of the optimal MnP and LiP activity, respectively. VP has a high affinity for H_2_O_2_, Mn^2+^, ferulic acid, -naphthol, and different hydroquinones and dyes, but their affinities for veratryl alcohol and substituted phenols are lower [13]. Veratryl alcohol, which increases the ability of LiP to oxidize high-redox-potential substrates, does not affect oxidation of these by versatile peroxidases of *B. adusta* and *P. eryngii*; these enzymes can oxidize several dyes that are not oxidized by lignin peroxidase in the absence of veratryl alcohol [17]. VPs form an attractive ligninolytic enzyme group due to their dual oxidative ability to oxidize Mn(II) and also phenolic and nonphenolic aromatic compounds, and could be used in programs for phytoremediation [18].

There have been many reports of phytoremediation using transgenic plants [19]. Transgenic plants expressing fungal laccases can remove Bisphenol A and pentachlorophenol from contaminated environments [20]. Previously, it has been reported that fungal Mn peroxidase-expressing transgenic tobacco was able to remove pentachlorophenol effectively [21].

In the present work, we generated transgenic tobacco plants that expressed the VP gene from *Bjerkandera adusta*, and showed that it can mitigate oxidative stress induced by paraquat, salt- (NaCl), drought- and osmotic-stress (sorbitol). These findings strongly suggest that the *VP* gene can be used as a tool to contend with different abiotic stresses. 

## 2. Results

### 2.1. Cloning and Sequence Analysis of the Bjerkandra adusta VP Gene

**Figure 1:**
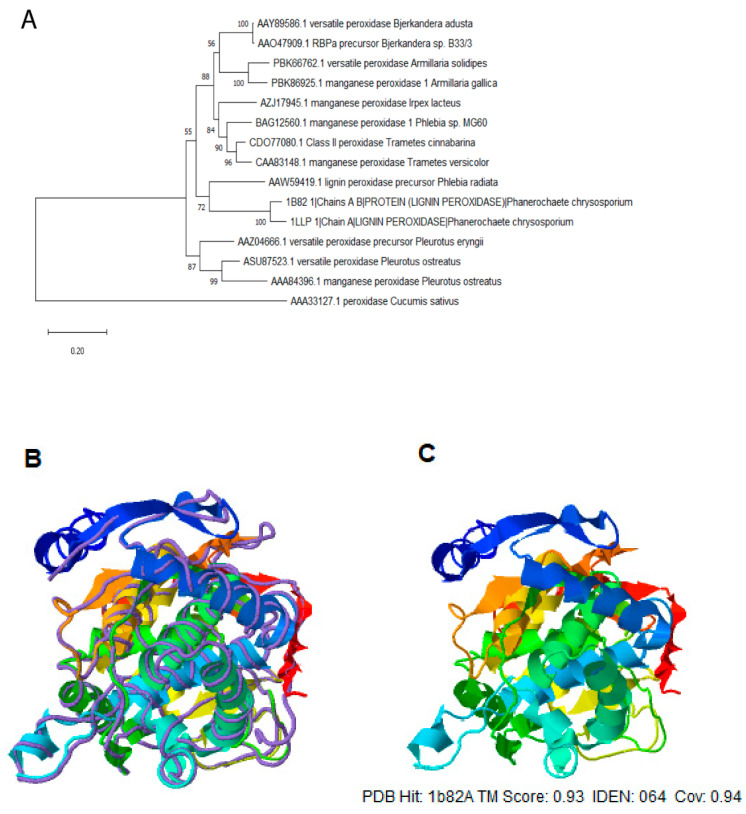
Phylogenetic relationships and protein models of versatile peroxidase from *Bjerkandera adusta*: (**A**) Analysis of versatile peroxidase with related sequences from GenBank was inferred using the neighbor-joining method, and the optimal tree is shown. The percentage of replicate trees in which the associated taxa clustered together in the bootstrap test (1000 replicates) are shown next to the branches. This analysis involved 15 amino acid sequences of fungal peroxidases. All ambiguous positions were removed for each sequence pair (pairwise deletion option). There was a total of 385 positions in the final dataset. Evolutionary analyses were conducted in MEGA X by using peroxidase AAA33127.1 (*Cucumis sativus*) as an outgroup. Scale bar refers to a phylogenetic distance of 0.2 amino acids substitutions per site. (**B**) Superposition of structure model VP (shown in cartoon) and lignin peroxidase (PDB ID: 1B82, shown in backbone), shows an overall similar helix structure. (**C**) A 3D structure model of VP was predicted by I-TASSER and visualized in Jmol. TM score: is a metric for assessing the topological similarity of protein structures, IDEN: is the percentage sequence identity in the structurally aligned region, Cov: represents the coverage of the alignment by TM-align and is equal to the number of structurally aligned residues divided by the length of the query protein.

To isolate a gene encoding a versatile peroxidase (VP) from *Bjerkandera adusta*, two oligonucleotides deduced from the *Bjerkandera* sp. strain B33/3 (GenBank accession No. AY217015) DNA sequence were used (see ‘Materials and Methods’), allowing the PCR amplification of a 1.1-kb DNA fragment cloned in pCR-BluntII-TOPO and designated pBVP02. DNA sequencing revealed a *VP* gene (Genbank accession No. DQ060037.1) and deduced protein (Genbank accession No. AAY89586.1), which shares 97 and 98.4% similarity to the *VP* gene and deduced protein respectively, from *Bjerkandera* sp. strain B33/3 [22]. The open reading frame encodes a protein of 366 amino acids with a calculated molecular mass of 38.4 kD (Figure S1) before predicted cleavage of a putative 21-amino-acid signal peptide (Figure S2). The alignment of the deduced amino acid sequences from related peroxidases showed several regions of homology and their secondary structure (Figure S3). To investigate the evolutionary relationships among VP in related fungi, a phylogenetic tree of the full-length amino acid sequences using the neighbor-joining method was constructed (Figure 1A) [23]. It can be observed that there are mainly two sequence homology groups. Firstly, two VP from the *Bjerkandera* genus are closely related to five manganese peroxidases from the *Armillaria* and *Trametes* genera, and they are less related to three lignin peroxidases from the *Phanerochaete* genus. In another homology group, two VP and one manganese peroxidase, all belonging to the *Pleurotus* genus, share sequence similarity. A third homology branch comprises a single distantly related plant peroxidase from cucumber. *VP* genes were only found in the fungi kingdom.

The predicted model of the VP structure (Figure 1B) includes 12 α-helices (Phe3-Gln20, Leu44-Phe57, Glu63-His74, Val114-Val125, Ala131-Cys147, Val178-Ala188, Thr193-Ala207, Thr229-Leu237, Gln263-Ala269, Ser271-Asn283, Gln285-Ile301, Ile338-Ala341), and two short beta-sheets 96 (Gly-Ile100, Glu51-Val52). Furthermore, it is observed to exhibit high structural identity when overlapped with lignin peroxidase (PDB ID: 1B82) from *Phanerochaete chrysosporium* (Figure 1C). In addition, we predicted the heme-binding residues (His66, Glu67, Leu69, Arg70, Phe73, Pro172, Glu173, Pro174, Ile181, Phe185, Leu199, Leu200, Ser202, His203, Ile205, Ala206, Ala207, Ala208, Asp209, His210, Val211, Phe220, Leu262, Ser264, Phe292, Met296), and the active site consisting of residues ARG70, HIS74, ASN111 (Figure S4).

### 2.2. Generation of Transgenic Tobacco Plants Expressing the VP Gene

**Figure 2:**
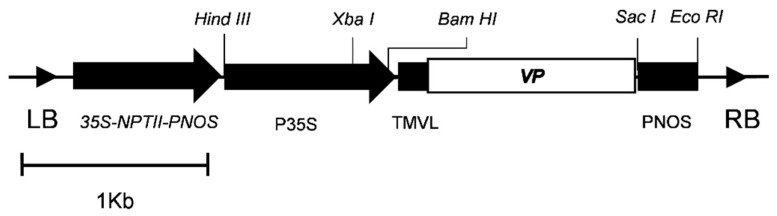
The T-DNA region of the Ti plasmid pBIN-VP. The cDNA encoding *Versatile Peroxidase* (*VP* gene) of *Bjerkandera adusta* UAMH8258 under the control of the CaMV 35S promoter (P35S) fused to the tobacco mosaic virus translation leader enhancer (TMVL) plus the nopaline synthase polyadenylation site (PNOS). The gene cassette is downstream of the kanamycin selectable marker *35S-NPTII-PNOS*, and between the left (LB) and right (RB) TDNA borders of *Agrobacterium tumefaciens* T-DNA in the pBin19 vector.

**Figure 3:**
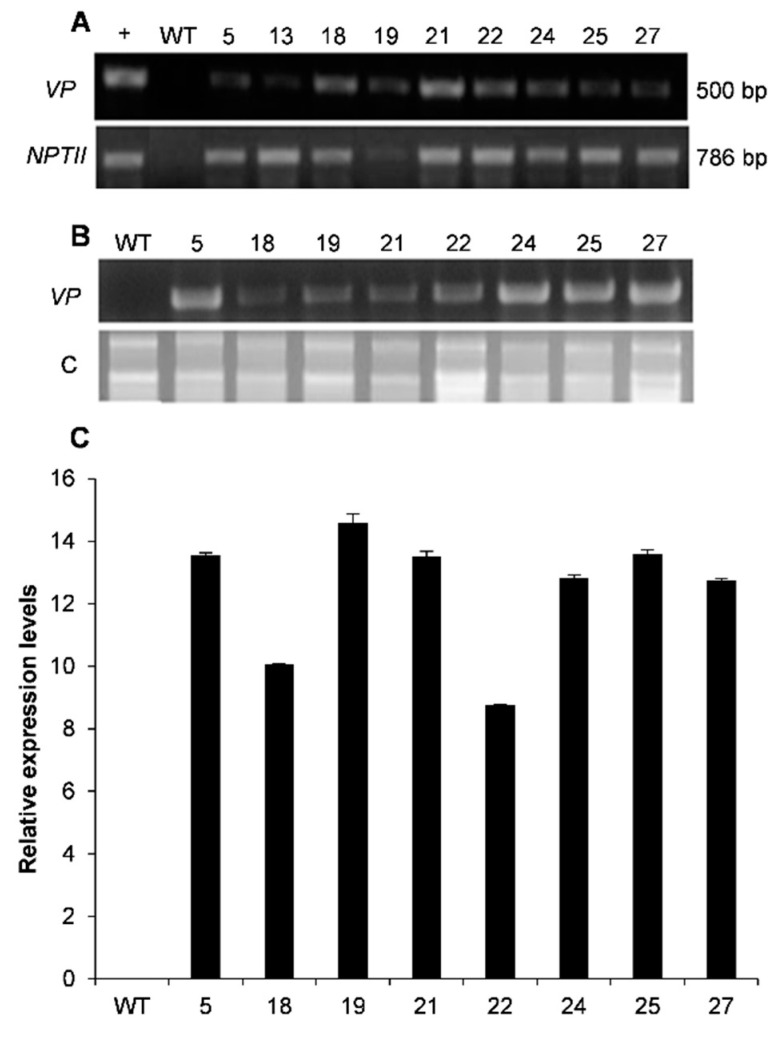
Molecular analysis of VP transgenic plants: (**A**) Top: positive control of *VP*-gene amplified from plasmid pBVP02 (see “Materials and methods”) (+), VP transgenic tobacco plants (5, 13, 18, 19, 21, 22, 24, 25, and 27), wild-type plants (WT) and plasmid (pBIN-VP) PCR-amplified product with VP Fwd and VP Rev primers; bottom: PCR-amplified product with NPTII Fwd and NPTII Rev primers. (**B**) Top: RT–PCR expression analysis in leaves amplified product with VP Fwd and VP Rev primers; bottom: Ribosomal RNA (rRNA) is used as an endogenous control gene (c). (**C**) Detection of transgenic tobacco VP lines by RT –PCR. Expression level of the *VP* gene in different transgenic lines (5, 18, 19, 21, 22, 24, 25, and 27) and WT plants.

Leaf discs from *N. tabacum* cv. Petit Havana were co-cultivated with *A. tumefaciens* LBA4404 harboring the construct pBIN-VP, which contains the coding sequence of the *B. adusta VP* gene, the CaMV35S promoter sequence fused to the TMVL to enhance protein translation [24], and the PNOS polyadenylation site, cloned in the pBin19 vector (Figure 2). During all stages of the regeneration of the transgenic plants, kanamycin was added to the media used for shoot and root generation, as detailed in the experimental procedure (Materials and Methods). A total of 30 putative T_0_ transgenic plants were obtained. Homozygous lines were identified on the selection medium containing 100 mg L^–1^ kanamycin in the T_2_ generation. Only 26 T_2_ lines survived the kanamycin selection by producing vigorous roots and shoots and are named VP lines. The presence of the *VP* foreign gene was confirmed with PCR. Nine putative transgenic plants were identified through genomic PCR-based screening of plants expressing *nptII* and *VP* genes (Figure 3A). The expression levels of *VP* transcript of the selected lines of transgenic plants were analyzed by reverse transcription PCR (RT–PCR) (Figure 3B). The expression of the *VP* transcript was observed in eight of the T_2_ transgenic plants with varied expression levels. Transgenic lines 18 and 22 showed moderate expression level, while relatively higher expression was found in plant lines 5, 19, 21, 24, 25, and 27 (Figure 3C).

### 2.3. Screening the Transgenic Plants for Higher MnP Activity

**Figure 4:**
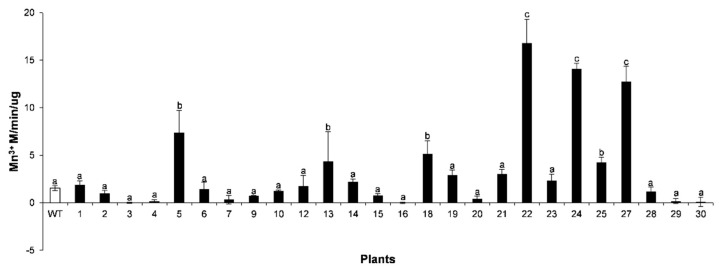
Specific manganese peroxidase activity in transgenic VP lines (1–30) and wild-type (WT) plants. The values represent the mean ± SD of three replicates from two separate experiments. Letters indicate significant differences in enzyme activity between transgenic (black bar) and WT (white bar) plants (Tukey’s test, *p* ≤ 0.05).

The 26 T_2_ generation lines were used for further characterization. Their Mn peroxidase (MnP) activities in leaf extract of VP22, VP24, and VP27 were 10.87-, 9.12-, and 8.24-fold higher than the control wild type, while VP5, VP18, and VP25 were 4.77-, 3.33-, and 2.75-fold higher, respectively; in contrast VP19 and VP21 were 1.90- and 1.94-fold higher than the control (Figure 4). *VP* gene expression ensures a significant increase of MnP activity in the leaf extract. The transgenic lines VP4, VP16, and VP20 had lower MnP activity than the control, probably due to T-DNA insertion in methylated or silenced regions in the transgenic genome. The VP8, VP11, VP17, and VP26 are absent from the graph because they had no progeny in the T_2_ generation.

**Figure 5:**
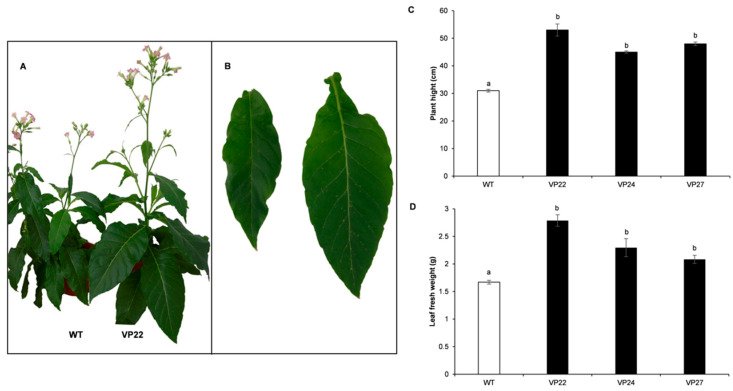
Enhanced plant growth in transgenic plants that overexpress the versatile peroxidase (VP) gene. (**A**) A representative VP line (VP22) and wild-type (WT) plants. (**B**) Leaves of VP22 and WT plants. Plant height (**C**) and leaves’ fresh weight (**D**) of six-week-old tobacco plants was determined in three independent experiments. Percentages were calculated taking the value of the WT strain as 100%. The white bar denotes WT plants, and black bars correspond to transgenic plants. Ten individuals for each line were taken for the statistical analysis. Letters indicate significant differences between transgenics (black bar) and WT (white bar) plant samples (Tukey’s test *p* < 0.05).

Hereafter, we selected the VP lines VP22, VP24, and VP27 that had higher MnP activities for futher analysis. Interestingly, the average stem length of transgenic tobacco plants expressing the *VP* gene is approximately 34% taller than the wild-type plants, and there is a 28% average increase in fresh weight after three months in greenhouse conditions (Figure 5). Several reports have documented that transgenic overexpression of peroxidase genes promote plant growth and faster development [25,26,27]. These results suggest that overexpression of *VP* in plants could be an effective strategy for enhancing biomass production.

### 2.4. Drought Tolerance in Transgenic Tobacco Plants

**Figure 6:**
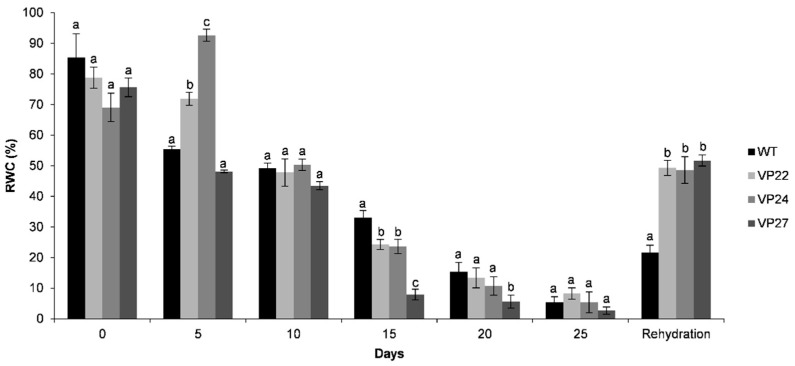
Overexpression of the *VP* gene increased drought tolerance in tobacco. Six-week-old tobacco plants were subjected to 25 days of dehydration and 7 days of rehydration treatments and thereafter the leaves were sampled to measure relative water content (RWC). The values represent the mean ± SD of three replicates from two separate experiments. Letters indicate significant differences in RWC between transgenic and wild-type (WT) plants (Tukey’s test, *p* ≤ 0.05).

**Figure 7:**
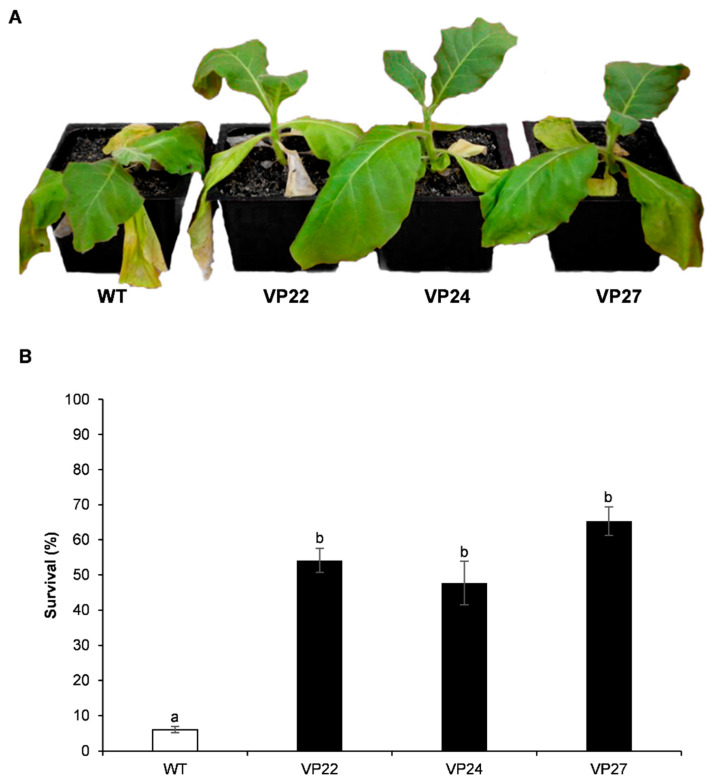
Effect of *VP* gene expression in drought tolerance assay in *N. tabacum*. (**A**) Transgenic and wild-type (WT) tobacco plants grown in pots were water-deprived for 25 days. Photograph was taken 7 days after rehydration. (**B**) The survival rate of plants was determined in 10 individuals per transgenic line or WT after a drought stress and recovery period, and two independent experiments were conducted. Letters indicate significant differences in RWC between transgenic (black bar) and WT plants (white bar) (Tukey’s test, *p* ≤ 0.05).

Relative water content (RWC) is a specific tool for the measurement of drought tolerance and gives a credible evaluation of the plant water status [28]. In fact, RWC estimates the deficit in water level at a given time, which is used to reflect dehydration stress. To investigate the relationship between dehydration stress and expression of the *VP* gene, RWC was measured in untreated six-week-old tobacco plants and dehydration-stress-induced plants at different times after treatment. Compared to the well-watered control, plants under dehydration stress displayed lower RWC when dehydration treatment increased from 20 to 25 days (Figure 6). After seven days of rehydration treatment VP22, VP24, and VP27 recovered turgor and the leaves were sampled to measure RWC. These three transgenic lines recovered from water deprivation after rewatering for seven days, whereas wild-type plants did not survive the dehydration treatment (Figure 7). After rewatering, transgenic plants continued their normal growth, flowering, and seed development.

### 2.5. Salinity and Osmotic Stress Tolerance in Transgenic Lines

**Figure 8:**
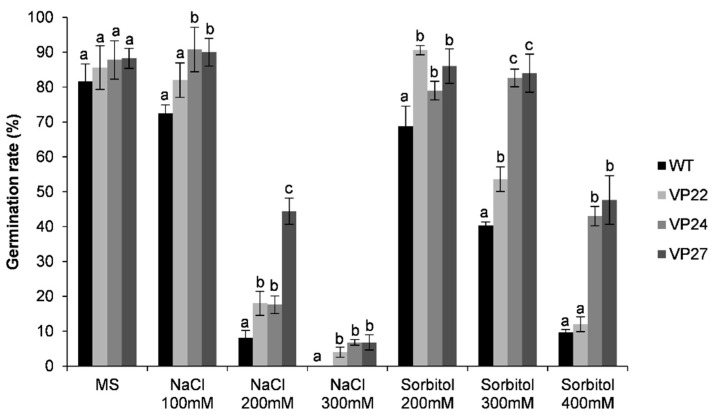
Seed germination rate in VP lines (22, 24, and 27) wild-type (WT) plants. Seeds were germinated at room temperature either on MS medium alone, containing 100, 200, and 300 mM NaCl, or 200, 300, and 400 mM sorbitol. Seed germination was evaluated after 13 days. The values represent the mean ± SD of five replicates from three separate experiments. Letters indicate significant differences in germination rate between transgenic and wild-type plants (Tukey’s test, *p* ≤ 0.05).

We analyzed seed germination in 13-day-old transgenic seedlings grown in the greenhouse in salt and osmotic stress assays. For assessing the salinity tolerance, germination of homozygous T_2_ seeds were studied on 100, 200, and 300 mM NaCl. The wild-type and transgenic seeds germinated normally on a MS medium without NaCl, although they showed variation on germination rate at different salinity levels. The VP22, VP24, and VP27 transgenic seedlings germinated on 100 mM NaCl at a comparable rate to MS without salt, whereas the wild-type seedlings germination rate decreased 16.5%. In contrast, in 200 mM NaCl the germination rate was severely affected and all seedlings showed chlorosis. Transgenic lines’ germination rate dropped to 80–50% on 200 mM NaCl compared to germination in 100 mM conditions. However, there were significant differences in seed germination between transgenic and wild-type seedlings on 200 mM NaCl. The VP27 line displayed a four-fold higher germination rate than wild-type seedlings, whereas VP22 and VP24 showed two-fold higher germination rates than wild-type seedlings on 200 mM NaCl stress (Figure 8). The VP27 line had a 2.5-fold higher germination rate than VP22 and VP24 lines. As the NaCl concentration increased, there was a significant reduction in germination rate in the wild type. After 13 days on 300 mM NaCl the transgenic seeds displayed an 8% germination rate, whereas the wild-type seeds did not show any germination at all (Figure 8). The transgenic seedlings also exhibited a stunted growth rate and chlorotic appearance on 300 mM NaCl.

The osmotic stress response of transgenic and wild-type plants was also examined at the seed germination level on media containing 200, 300, and 400 mM sorbitol. The transgenic lines grew normally in 200 mM sorbitol compared to the seedling grown in MS media without sorbitol (Figure 8), however the wild-type seedlings showed a 20% decrease in germination rate compared to the VP22 and VP27 lines. At 300 mM sorbitol, VP24 and VP27 could still germinate at a comparable rate with seedlings grown on 200 mM and wild-type seedlings without sorbitol (Figure 8). In contrast, on 400 mM sorbitol the germination rate of transgenic VP24 and VP27 lines decreased 50% compared to their growth on 300 mM sorbitol, whereas VP22 and wild-type seedlings showed only a 10% germination rate (Figure 8).

### 2.6. Oxidative Stress Effect on Transgenic Tobacco

**Figure 9:**
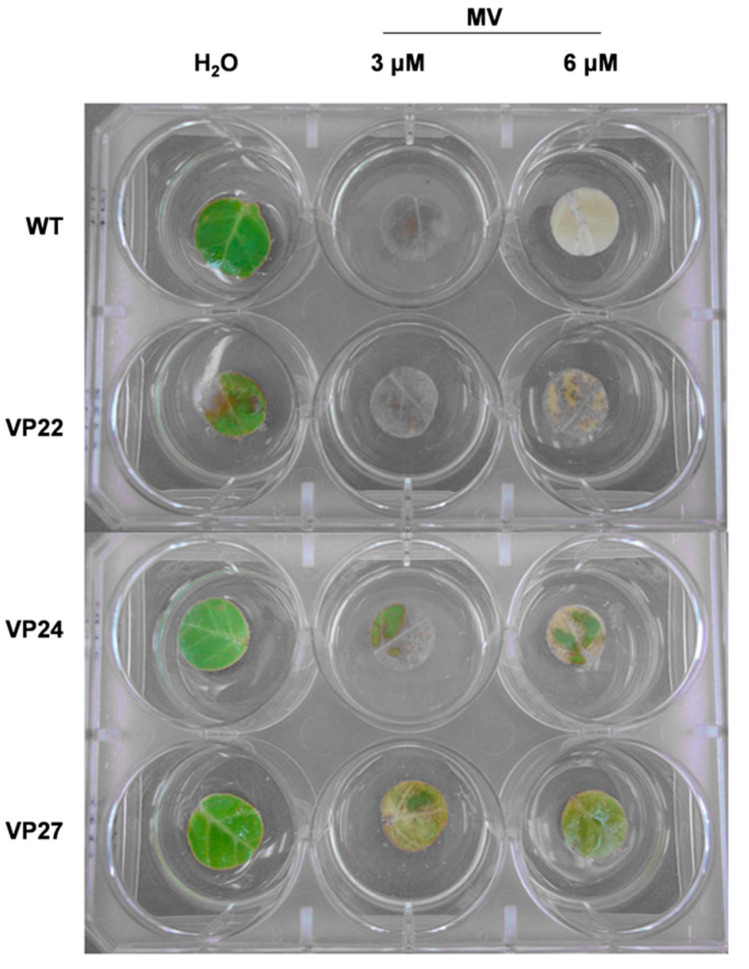
Enhanced oxidative stress tolerance of VP lines (22, 24 and 27) and wild-type (WT) plants grown under greenhouse conditions. Representative photograph of leaf samples floated on distilled water, 3 and 6 µM of methyl viologen (MV) for 3 days under light conditions.

**Figure 10:**
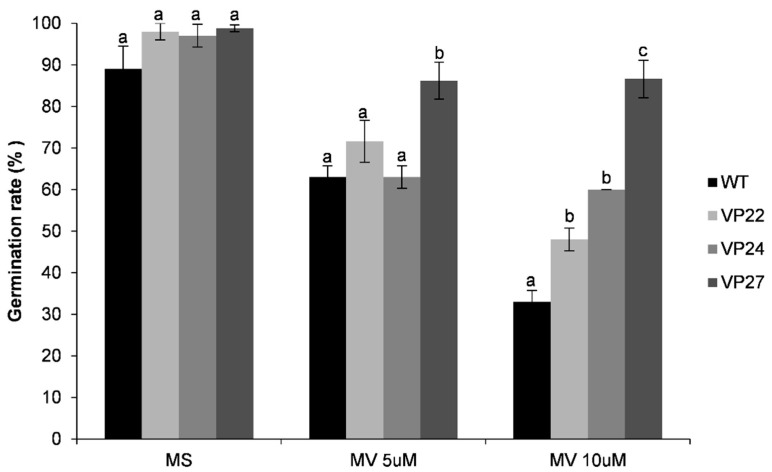
Oxidative stress tolerance in seed germination rate in 35S::VP lines (VP22, VP24, and VP27) and wild-type (WT) plants. Seeds were germinated at greenhouse conditions either on MS medium alone or containing 5 and 10 µM methyl viologen (MV). Seed germination was evaluated after 15 days. The values represent the mean ± SD of five replicates from three separate experiments. Letters indicate significant differences in germination rate between transgenic and wild-type plants (Tukey’s test, *p* ≤ 0.05).

We investigated whether the transgenic plants could survive the oxidative stress generated by MV (methyl viologen). The visible injury was presented in Figure 9. The V24 and VP27 transgenic lines survived to 3 and 6 µM MV compared to the VP22 transgenic line and the wild type (Figure 9). When the effect of 5 and 10 µM MV on germination rate was analyzed, it showed that the transgenic plants were more tolerant than the wild type on 10 µM MV (Figure 10). Therefore, the VP overexpression in tobacco caused an enhanced tolerance to oxidative stress generated by MV.

## 3. Discussion

Climate change and extensive agriculture practice have provoked water scarcity, severe droughts, and soil salinity around the world. Therefore, major efforts are required to improve abiotic stress tolerance in plants, either by classic genetics or genetic manipulation techniques using transgenic, CRISPR/Cas9, or oligonucleotide-directed mutagenesis tools [29]. A long list of transgenic plants with a myriad of exogenous genes has been shown to display an improved tolerance to drought, salt, cold, heat, and oxidative stresses [30]. Among these selected genes, peroxidases have been shown to enhance stress tolerance [26,27]. Plants have a large number of peroxidases isozymes, which are encoded by multigenics families. Plant peroxidases have several physiological functions, such as removing reactive oxygen species (ROS), biosynthesis and degradation of lignin in cell walls, auxin catabolism, defense response wilt, and defense against pathogens or insect attack [31]. However, most fungal peroxidases are extracellular enzymes whose major role is lignin degradation so as to allow the fungus to access cellulose and hemicellulose as carbon sources [14].

Our results showed the isolation of a novel versatile peroxidase (VP) from the white-rot fungus *Bjerkandera adusta*, since it has a unique evolutionary relationship with other fungal peroxidases (Figure 1). We analyzed the abiotic stress tolerance phenotype of transgenic tobacco plants overexpressing it. Up to now, these enzymes have only been found in fungi and, as far as we know, there are no reports of their transgenic overexpression in plants. Thus, we generated transgenic tobacco plants, which expressed the heterologous gene at high levels (Figure 3) and some lines showed significant Mn peroxidase (MnP) enzyme activity (Figure 4).

Several studies have shown various altered phenotypes caused by different peroxidases expressed in transgenic plants. For instance, transgenic alfalfa plants expressing the gene MnP of *Phanerochaete chrysosporium* expressed under the 35S promoter exhibited a dwarf phenotype compared with wild plants [32]. In contrast, the transgenic plants of our study showed no adverse effects in terms of growth, flowering, and seed production using the 35S promoter (Figure 5). Moreover, our VP transgenic lines have a significant increase of plant height and biomass as a result of *B. adusta VP* gene expression. This phenotype could be due to the loosening of the plant cell wall by partial lignin modification, allowing a larger cell growth. These traits are of great potential in forestry and paper and textile industries, suggesting that the *VP* gene could be used in transgenic trees to increase their wood content. Peroxidases are also associated with the process of lignification during the last step of deposition monolignol [33]. Many studies have investigated the modification of lignin content in transformed plants. Modifying the expression of genes involved in lignin biosynthesis, such as phenylalanine ammonia-lyase, cumaronil CoA reductase, and caffeoyl-CoA O-methyltransferase have been reported to improve wood quality for applications in paper, textile, fodder, and bioenergy industries, since the lignin matrix is probably modified and allows a better processing of the wood [34]. Additionally, it has been shown that the VP enzyme can oxidize diverse aromatic compounds and dyes, thus it can be speculated that a transgenic plant overexpressing the *VP* gene could potentially be used for phytoremediation as it has been shown with other fungal peroxidases and laccases expressed in plants [17,18,19,20,21].

The present work showed that production of the versatile peroxidase (VP) in tobacco plants improves drought, salt, and osmotic stress tolerance (Figure 6, Figure 7 and Figure 8). The most tolerant VP lines under salt, water, and sorbitol stress correlate with the higher VP activity in the transgenic plants. These findings agree with other reports where enhanced peroxidase activity in plants exposed to abiotic stress was observed [26,27,35,36]. Recently, a *Glycine soja GsPRX9* gene encoding a Class III peroxidase was overexpressed in soybean leading to enhanced salt tolerance and an antioxidant response [37]. The role of ascorbate peroxidase (APX) in stress tolerance was demonstrated in APX-antisense or APX-RNAi transgenic plants, which are highly susceptible to oxidative injury, whereas the overexpression of APX and other enzymes responsible of ascorbate biosynthesis confer drought, salt, high light, SO_2_, and ozone tolerance [38]. Also, it has been shown the relevance of overproduction of *Arabidopsis thaliana* cytosolic APX in *Nicotiana tabacum* chloroplast, to protect the plant from oxidative stresses generated by paraquat (methyl viologen), Na_2_SO_3_, salt stress (NaCl), drought stress, and polyethylene glycol-induced (PEG) stress [27].

In the present study, we also examined the oxidative stress with methyl viologen (MV) injury in tobacco plants overexpressing the *VP* gene. The leaf discs from the WT plants bleached completely with increasing concentrations of MV, whereas the transgenic plants showed partial signs of chlorosis suggesting that the transgenic plants obtained in this work are oxidative-stress tolerant (Figure 9). ROS detoxification for cell survival is partly achieved by peroxidases (3). Modification of the ROS scavenging systems of chloroplast can result in tolerance to oxidative stresses imposed by many biotic and abiotic factors in combination (cross-tolerance).

Additionally, we assessed the germination rate of VP lines under exposure to MV (Figure 10). Transgenic seeds displayed a significant increase in germination rate compared to WT plants. All together these results strongly support that the VP gene confers oxidative stress tolerance. Similar results were shown in cytosolic ascorbate peroxidase from spinach [39]. Class I ascorbate peroxidase is ubiquitous in higher plants, and its physiological function is to capture chloroplast H_2_O_2_ [40]. Cytosolic ascorbate peroxidase plays an important role in eliminating H_2_O_2_ and minimize photoxidative damage. Among the various functions of peroxidases is the control of cell growth using H_2_O_2_ as an electron acceptor [41]. VP belongs to Class II peroxidases, which are exclusively found in fungi as well as lignin peroxidases (LiP) that catalyze depolymerization of lignin, and in manganese peroxidases (MnP) responsible for the peroxide-dependent oxidation of Mn (II) to Mn (III), which serves as diffusible redox mediator [31]. In contrast, Class III peroxidases are widely distributed in plants and play key roles, such as cell wall metabolism, lignification, suberization, auxins metabolism, ROS detoxification, and defense against pathogen attack [42].

It is possible that the VP enzyme in transgenic tobacco has a larger redox potential than the plant peroxidases, allowing it to scavenge more efficiently ROS induced by MV. Also, a larger redox potential in plants could probably protect them from pathogen attack. [31]. Overexpression of a peroxidase in transgenic plants can increase the disease resistance of plants. For example, the overexpression of *HvPrx40* [43] leads to higher resistance to *Blumeria graminis* (wheat powdery mildew) in barley (*T. aestivum*). Future work might confirm whether the transgenic tobacco plants generated in the present study could resist pathogen attack.

ROS have also a role as secondary messengers inducing plant response to biotic and abiotic stresses [3]. It is tempting to speculate that VP might control plant growth and development through the H_2_O_2_ signal transduction pathway. H_2_O_2_ is a signaling molecule that regulates the expression of many transcription factors, including ZAT zinc finger transcription factor, WRKY, heat shock transcription factor, and ethylene response factor [44]. Moreover, ROS induce post-translational modifications such as sulfonylation and seem to regulate transcription factors machinery to adjust plants to an effective and systemic acclimation response [4].

## 4. Materials and Methods

### 4.1. Construction of a cDNA Library from Bjerkandra adusta

For cDNA library construction the *Bjerkandera adusta* UAMH825 strain was grown during 8 days at 28 °C with vigorous shaking in 3% bran flakes medium (60 mM phosphate buffer, pH 6.0). Total RNA was extracted with Trizol (ThermoFisher Scientific, Carlsbad, CA, USA) from 200 mg *Bjerkandera adusta* micellium. The cDNA library was constructed with 1 µg of mRNA using the BD Creator^TM^ SMART^TM^ cDNA Library kit (Clontech/Takara Bio Inc, USA). For *VP* gene isolation a PCR reaction with primers Fwd-EB001 5′-AATGGCCTTCAAGCAACTC-3′ and Rev-EB002 5-′GTCCCGCCTTCGTAACCTAGGATTCG-3′ containing a *Avr*II site (underlined) was conducted with the following program: one cycle at 95 °C, 5 min; and 30 cycles of 95 °C, 0.5 min; 62 °C, 0.5 min; and 72 °C, 1.5 min; and one last cycle at 72 °C, 10 min, using 20 ng DNA plasmid template extracted by the alkaline lysis method from the whole cDNAs library. The resulting PCR band was cloned in pCR-BluntII-TOPO leading to pBVP02 clone and sequenced.

### 4.2. Sequence Analysis and Protein Modelling

#### 4.2.1. Phylogenetic Analysis of Versatile Peroxidase

The phylogeny was inferred using the neighbor-joining method [23]. The optimal tree is shown. The percentage of replicate trees in which the associated taxa clustered together in the bootstrap test (1000 replicates) are shown next to the branches. The tree is drawn to scale, with branch lengths in the same units as those of the evolutionary distances used to infer the phylogenetic tree. The evolutionary distances were computed using the Poisson correction method and are in the units of the number of amino acid substitutions per site. This analysis involved 16 amino acid sequences. All ambiguous positions were removed for each sequence pair (pairwise deletion option). There was a total of 385 positions in the final dataset. Evolutionary analyses were conducted in MEGA X [45] by using peroxidase AAA33127.1 (*Cucumis sativus*) as an outgroup.

#### 4.2.2. Structural and Modeling of Versatile Peroxidase (VP)

The prediction of the PV structural model and structure-based function annotation was performed using the I-Tasser program [46]. I-TASSER (Iterative Threading ASSEmbly Refinement) is a hierarchical approach to protein structure prediction and structure-based function annotation [47]. It first identifies structural templates from the PDB by the multiple threading approach LOMETS, with full-length atomic models constructed by iterative template-based fragment assembly simulations. Function insights of the target are then derived by re-threading the 3D models through the protein function database BioLiP [48].

### 4.3. Construction of a Binary Vector with Versatile Peroxidase Gene

The 1.1-kb *VP* gene from pBVP02 clone was amplified by PCR using primers VPup 5´-CGCGGATCCATGGCCTTCAAGCAACTC-3´ containing a *Bam*HI site and VPdown, 5´-CGAATGAATTCGAGCTCTTACGAAGGCGGGAC-3´ with *Eco*RI and *Sac*I sites (underlined), using 100 ng of linearized pCR-Blunt II-TOPO-VP plasmid DNA containing the *B. adusta VP* gene, the *Pfu* DNA polymerase (Fermentas Thermo Scientific, USA) and the following conditions: one cycle at 94 °C, 5 min; and 35 cycles of 94 °C, 1 min; 55 °C, 1 min; and 72 °C, 1.5 min; and one last cycle at 72 °C, 5 min. Then, the PCR fragment was subcloned in the *Bam*HI and *Eco*RI sites of the pKSJLΩ27 vector containing the 78-bp leader of tobacco mosaic virus translational enhancer (TMVL) [24]. Finally, the TMVL-cDNA fusion was subcloned into the binary vector pBin19 containing 35S promoter and the NOS polyadenylation site, with *Xba*I and *Sac*I to generate the pBIN-VP construct.

### 4.4. Transformation of Tobacco Plants

The plasmid construct which carries the *nptII* gene to allow kanamycin selection was mobilized into *Agrobacterium tumefaciens* LBA4404 by electroporation. The T-DNA region of pBinVP was introduced into the genome of *Nicotiana tabacum* cv. Petite Havana, by the leaf-disk method using *A. tumefaciens* [49]. These transgenic tobacco plants, which have regenerated from leaf disks and were resistant to kanamycin, were transferred to Murashige–Skoog (MS) medium containing 30 g L^−1^ sucrose and 100 mg L^−1^ kanamycin and 500 mg L^−1^ carbenicillin, and then incubated in a growth chamber at 25 °C and a photoperiod of 16 h light and 8 h dark. The integration of T-DNA into the tobacco genome was confirmed by PCR with primers designed to amplify the introduced *VP* cDNA.

### 4.5. Genomic DNA Analysis

Genomic DNA from tobacco was isolated with Gentra Puregene DNA isolation kit (Quiagen, Hilden, Germany) and 100 ng were used for a polymerase chain reaction (PCR) using oligonucleotides corresponding to neomycin phosphotransferase (*nptII*) gene (5′-GAACAAGATGGATTGTTGCACGC-3′ and 5′-GAAGAACTCGTCAAGAAGC-3′). The program was: one cycle at 94 °C for 5 min; 30 cycles at 94 °C for 30 s, 59 °C for 30 s, and 72 °C for 45 s; and finally, one cycle at 72 °C for 5 min. Moreover, a 1.1-kb *VP* gene fragment was amplified by PCR using primers VPup and VPdown, as described above.

### 4.6. Gene Expression Analysis

Reverse transcription polymerase chain reaction experiments were performed using 2 μg of total RNA extracted from tobacco adult plants (5, 18, 19, 21, 22, 24, 25, 27, and WT) using TRIzol reagent (Thermo Fisher Scientific, Carlsbad, CA) according to the manufacturer’s instructions and used for first-strand cDNA synthesis with SuperScript II reverse transcriptase (Thermo Fisher Scientific, Carlsbad, CA) and oligo dT. The polymerase chain reaction was conducted using oligonucleotides corresponding to 0.5 kb into the *VP* gene, VPFwd2 5′-CGACCCGACGATCCCTGGAT-3′ and VPPrev2 5′-CACAATTCCTACGACGACGC-3′, and the program was as described above, except that annealing was at 62 °C and only 20 cycles were used, which corresponded to the linearity phase of the exponential reaction after comparison of the PCR products at different cycles. Polymerase chain reaction products were resolved in 1X TAE and 1% agarose gels, and stained with ethidium bromide (EtBr). Furthermore, qRT-PCR was performed using the fluorescent intercalating dye SYBR Green Master Mix (Invitrogen, USA). The qRT-PCR was performed using BjVPfwd 5′-ATGGCCTTCAAGCAACTCCT-3′ and VPLOWqRT 5- TCGCCGCCGTCGAACAAGT-3′ primers that amplify a 180 bp fragment. The actin gene was selected as internal control; we used NtACT-Fwd 5′- AAGGTTACGCCCTTCCTCAT-3′ and NtACT-Rev 5′-CATCTGTTGGAAGGTGCTGA-3′ with 566 bp product; PCR conditions were 50 °C for 2 min, 95 °C for 2 min, and 95 °C 15 s, 60 °C 1 min 40 cycles. All reactions—technical and biological—were performed in triplicate. The CT values were calculated by the 2-ΔΔCT method [50].

### 4.7. Enzyme Assays

Protein extraction was performed at 4 °C from leaves of primary transformants and wild-type tobacco plants. Plant tissue (2 mg leaf piece) was macerated in a grinder with 50 mM malonate buffer (pH 4.5) adding 25 µL mL^−1^ phenylmethylsulfonyl fluoride (PMSF), then transferred to a microtube and the mixture was centrifugated for 10 min at 13,000 rpm at 4 °C. Afterwards 0.1 % polyvinylpyrrolidone (PVP) was added to the supernatant, mixed with vortex for 20 s, and finally centrifuged for 10 min at 13,000 rpm at 4 °C before being assayed for enzyme activity. Manganese peroxidase (MnP) activity was measured. MnP activity was routinely measured by the H_2_O_2_-dependent formation of Mn (III) malonate complex at 270 nm (ε = 11,590 M^−1^cm^−1^). Reactions contained 1 mM manganese sulfate in 50 mM malonate buffer (pH 4.5), and the reaction was initiated by the addition of 0.1 mM H_2_O_2_ [51].

### 4.8. Relative Water Content Assay

Seeds of three homozygous transgenic (T2) lines VP22, VP24 and VP27 were surface sterilized with 100% C_2_H_6_O during 1 min, and with 10% NaClO and 0.2% Tween for 5 min, washed 5 times with distilled sterile water, and germinated on MS medium with kanamycin (100 mg L^−1^). Seedlings in Petri dishes were maintained in a culture room at 27 ± 1 °C with a photoperiod of 16 h light and 8 h dark for 2 weeks. Seedlings from each of the three transgenics (VP22, VP24, and VP27) and wild-type plants were transferred to pots with vermiculite and grown in a greenhouse with a photoperiod of 16 h light and 8 h dark conditions for 4 weeks. The homozygous six-weeks-old tobacco plants were subjected to 15 days of dehydration and seven days of rehydration treatment and the leaves were sampled to measure relative water content (RWC). Five individuals from wild-type plants and each transgenic line used in the present study were taken and weighed immediately after leaf detaching (fresh weight). Plant RWC and their fresh weight was determined from excised leaves. After floating them in deionized water at 4 °C overnight, their rehydrated weight was determined. The dried weight was determined after incubating leaves in an oven at 70 °C during 24 h. Plant RWC (%) was calculated by measuring (fresh weight–dry weight)/(rehydrated weight–dry weight) × (100) [28].

### 4.9. Germination Analysis of Transgenics for Abiotic Stress Tolerance

Seeds of wild-type and homozygous transgenic plants (VP22, VP24, and VP27) were sterilized and germinated on normal MS medium and MS containing 100, 200, and 300 mM NaCl or 200, 300, and 400 mM sorbitol. Also, 5 and 10 µM methyl viologen concentrations to germination oxidative stress treatment. The seeds were grown in a growth chamber at 25 ± 1 °C and a photoperiod of 16 h light and 8 h dark. 

### 4.10. Oxidative Stress Experiments

Leaf discs (3.0 cm diameter) were excised from the healthy and fully expanded tobacco leaves of 6-week-old wild-type and transgenic plants with higher Mn peroxidase activity (VP22, VP24, and VP27) from the greenhouse using a cork borer. Leaf discs were floated on 2 mL of the respective methyl viologen (MV) concentrations (0, 3.0 and 6.0 µM) and distilled water, before being incubated under greenhouse conditions for 72 h and a photoperiod of 16 h light and 8 h dark [27].

### 4.11. Statistical Analysis

Data analyses were performed using analysis of variance (ANOVA) with the SAS-University-Edition program [52]. All data are presented with 3–5 replicates of two or three independent experiments, and comparison of means was determined by Tukey’s test (*p* ≤ 0.05).

## 5. Conclusions

Organisms have developed an efficient antioxidant machinery, including peroxidases among other enzymes, that help to maintain cellular homeostasis by counteracting ROS. The results presented in this study provide evidence that transformed tobacco plants with a chimeric *VP* gene displayed reduced damage to drought stress, and treatments up to 400 mM sorbitol, 200 mM NaCl, and 10 µM MV. Although more work is needed to understand the exact mechanism involved in the VP enzyme leading to an increase in plant biomass, and protection against drought, salt, osmotic, and oxidative stresses in transgenic plants, it is at least in part the consequence of the VP MnP activity, which displays a redox potential capacity to counteract ROS damage. As far as we know, this is the first report of transgenic plants tolerant to abiotic stress with a *VP* gene, and supports the hypothesis that genetic manipulation of stress tolerance in crops using Versatile Peroxidase could be an important agro-biotechnological tool in the future. 

## Data Availability

The data presented in this study are available on request from the corresponding author.

## Supplementary Materials

https://www.mdpi.com/article/10.3390/plants10050859/s1. Figure S1. Amino acid sequence of versatile peroxidase from *Bjerkandera adusta* (Genbank accession No. AAY89586). Signal peptide amino acid residues are blue underlined. Active site amino acid residues are H74 y H203 (red bold); residues interacting with heme group: H66, E67, L69, R70, F73, W198, A201, A206, A207, A208, D209, H210, V211, 262 Y Q264 (blue shaded). According to ExPASy, VP with signal peptide has pI 4.41, MW 38384.09 Da and 366 amino acids and VP without signal peptide has pI 4.36, MW 36239.48 Da and 345 amino acids. Figure S2. Signal peptide prediction site of VP. Signal peptide prediction (Sec/SPI) tool in P-5.0 analysis website predicts the cleavage site between amino acid positions 21 and 22: SQA-AI. Probability: 0.8116. Figure S3. Secondary structure analysis of versatile peroxidase and its homologs amino acid sequences using EsPript3 program. (XP_036630848.1, *Pleurotus ostreatus* Versatile peroxidase vpl1; AAZ04666.1, *Pleurotus eryngii* versatile peroxidase precursor; ASU87523.1, *Pleurotus ostreatus* versatile peroxidase; PBK66762.1, *Armillaria solidipes* versatile peroxidase; CDO77080.1, *Trametes cinnabarina* Class II peroxidase; AAY89586.1, *Bjerkandera adusta* versatile peroxidase; and AAO47909.1, *Bjerkandera* sp. B33/3 RBPa precursor). Catalytic site residues are depicted with a red triangle and residues interacting with Heme group are marked as blue squares. Figure S4. Prediction of ligand binding sites and active site. (A) Ligand binding sites were predicted according to the COFACTOR and COACH servers. The best hit model was the heme group of the protein 2gb8A (PDB ID: 2GB8, Cytochrome c peroxidase) with a C-score value of 0.93. Ligand binding specifics residues are: His66, Glu67, Leu69, Arg70, Phe73, Pro172, Glu173, Pro174, Ile181, Phe185, Leu199, Leu200, Ser202, His203, Ile205, Ala206, Ala207, Ala208, Asp209, His210, Val211, Phe220, Leu262, Ser264, Phe292, Met296. (B) Catalytic sites were predicted by comparison of the VP structural model and the *Coprinus cinerea* oxidoreductase (PDB ID: 1LY8, C-core Enzyme Commission= 0.57, TM score = 0.91). Active sites residues are: Arg70, His74, Asn111.
