# Supplementary material for: A Versatile Peroxidase from the Fungus Bjerkandera adusta Confers Abiotic Stress Tolerance in Transgenic Tobacco Plants
Source: Plants (Basel). 2021 Apr 23;10(5):859. doi: 10.3390/plants10050859 (PMC8146367; doi:10.3390/plants10050859)
Supplement: Supplementary file 1 [file plants-10-00859-s001.zip › plants-1182923-supplementary.pdf]

Supplementary Materials

*MAFKQLAAALSIALALPFSQA*AITRRVACPDGVNTATNAACCALFAVRDDIQQ

NLFDGGECGEEVHESLRLTF**H**DAIGISPSIAATGKFGGGGADGSIMIFDDIEPNFH

ANNGVDEIISAQKPFVAKHNMTAGDFIQFAGAVGVSNCPGAPQLSFFLGRPAAT

QPAPDGLVPEPFDSVTDILNRFADAGGFTTQEVVWLLAS**H**SIAAADHVDPTIPGS

PFDSTPEIFDTQFFVETLLKGTLFPGTSGNQGEVESPLAGEIRLQSDADFARDSRTA

CEWQSFVNNQPRMQVLFKAAMQKLSILGHDLTQMIDCSDVIPVPPSTAVRGSHL

PAGNTLDDIEQACASTPFPTLTADPGPATSVAPVPPS

Figure S1. Amino acid sequence of versatile peroxidase from *Bjerkandera adusta* (Genbank accession No. AAY89586). Signal peptide amino acid residues are blue underlined. Active site amino acid residues are H74 y H203 (red bold); residues interacting with heme group: H66, E67, L69, R70, F73, W198, A201, A206, A207, A208, D209, H210, V211, 262 Y Q264 (blue shaded). According to ExPASy, VP with signal peptide has pI 4.41, MW 38384.09 Da and 366 amino acids and VP without signal peptide has pI 4.36, MW 36239.48 Da and 345 amino acids.

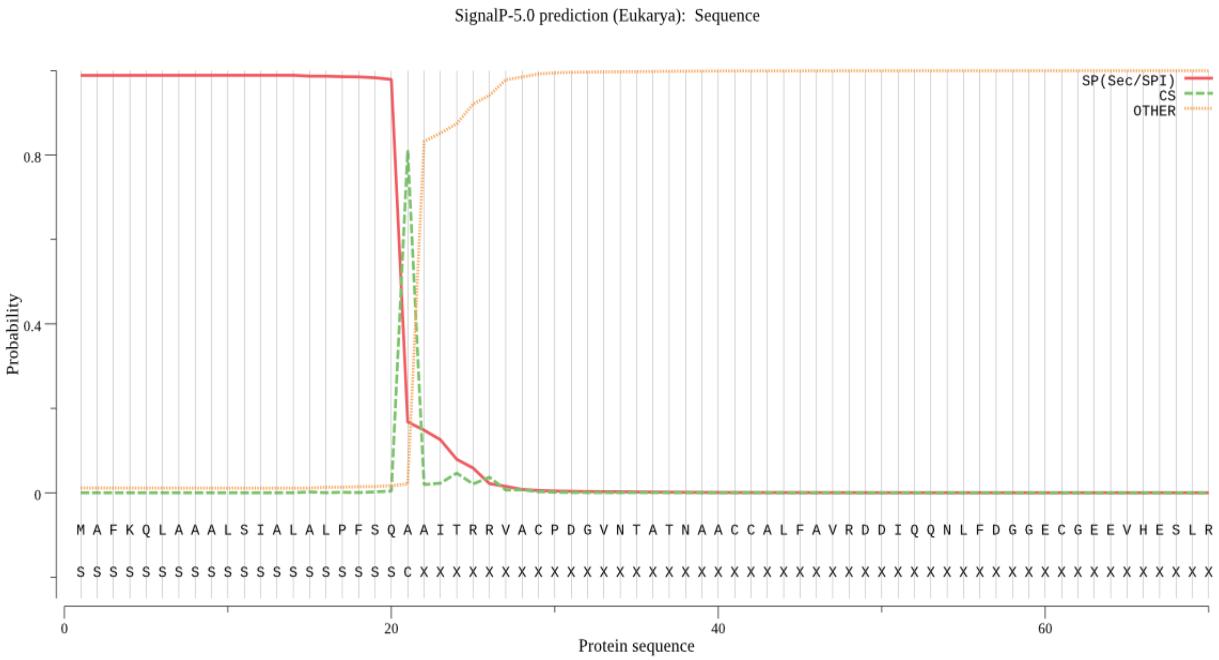

Figure S2. Signal peptide prediction site of VP. Signal peptide prediction (Sec/SPI) tool in P-5.0 analysis website predicts the cleavage site between amino acid positions 21 and 22: SQA-AI. Probability: 0.8116.

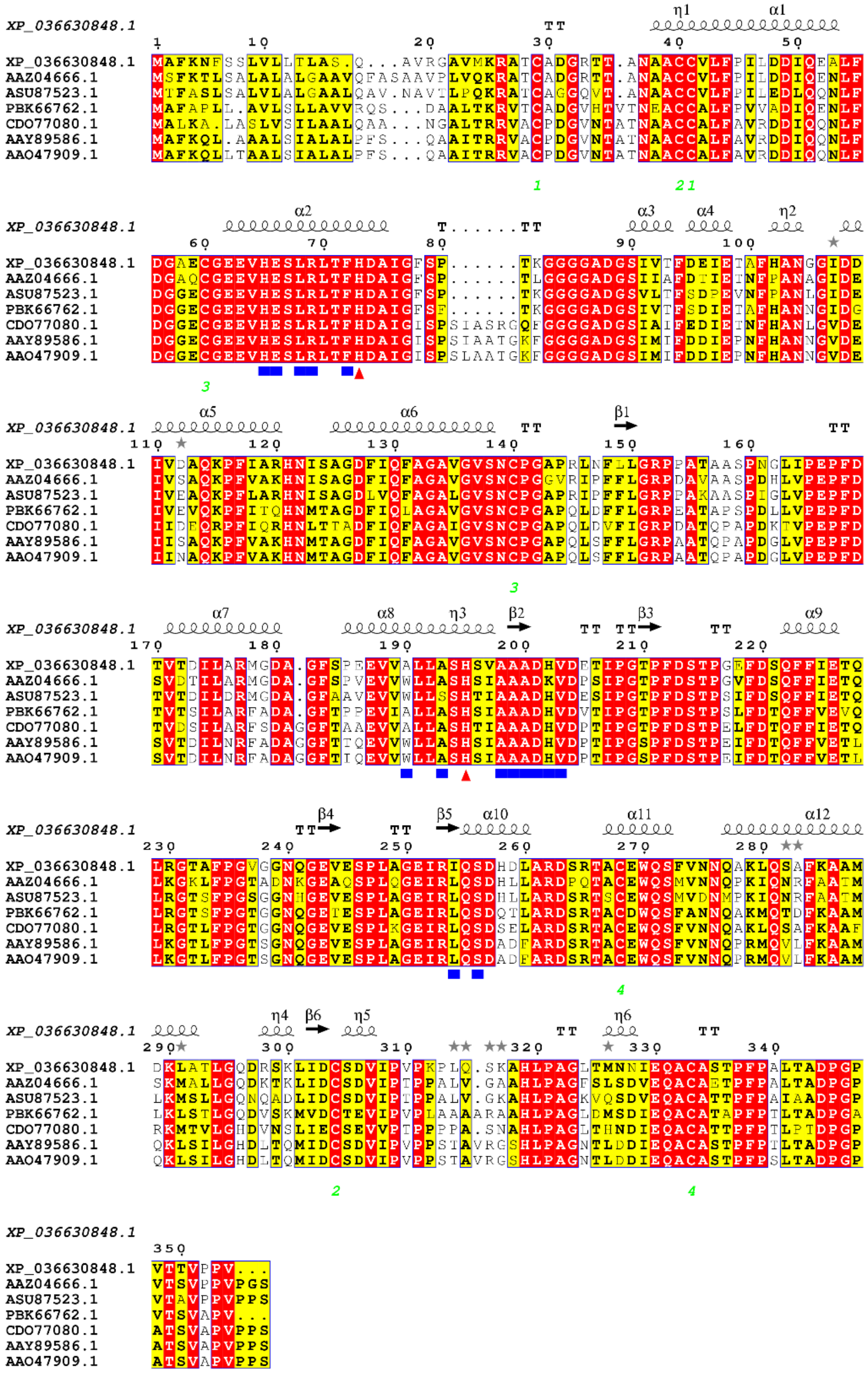

Figure S3. Secondary structure analysis of versatile peroxidase and its homologs amino acid sequences using EsPript3 program. (XP_036630848.1, *Pleurotus ostreatus* Versatile peroxidase vpl1; AAZ04666.1, *Pleurotus eryngii* versatile peroxidase precursor; ASU87523.1, *Pleurotus ostreatus* versatile peroxidase; PBK66762.1, *Armillaria solidipes* versatile peroxidase; CDO77080.1, *Trametes cinnabarina* Class II peroxidase; AAY89586.1, *Bjerkandera adusta* versatile peroxidase; and AAO47909.1, *Bjerkandera* sp. B33/3 RBPa precursor). Catalytic site residues are depicted with a red triangle and residues interacting with Heme group are marked as blue squares.

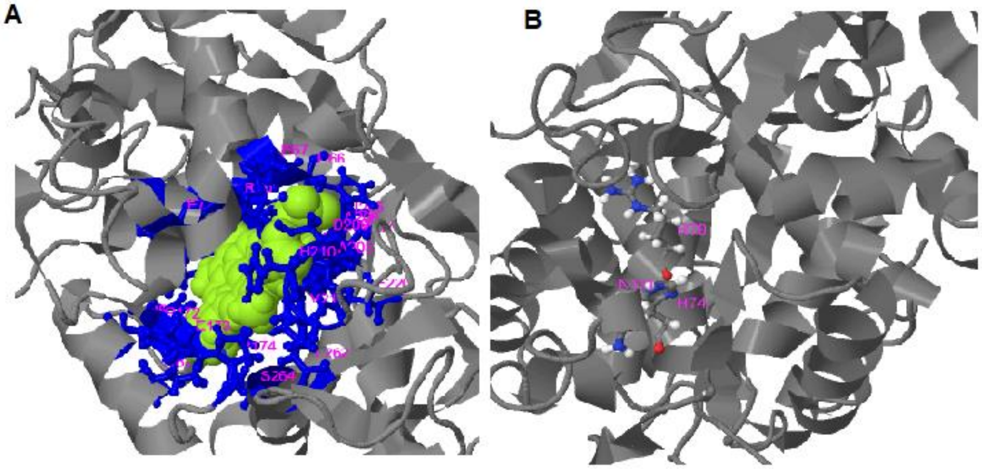

Figure S4. Prediction of ligand binding sites and active site. (A) Ligand binding sites were predicted according to the COFACTOR and COACH servers. The best hit model was the heme group of the protein 2gb8A (PDB ID: 2GB8, Cytochrome c peroxidase) with a C-score value of 0.93. Ligand binding specifics residues are: His66, Glu67, Leu69, Arg70, Phe73, Pro172, Glu173, Pro174, Ile181, Phe185, Leu199, Leu200, Ser202, His203, Ile205, Ala206, Ala207, Ala208, Asp209, His210, Val211, Phe220, Leu262, Ser264, Phe292, Met296. (B) Catalytic sites were predicted by comparison of the VP structural model and the *Coprinus cinerea* oxidoreductase (PDB ID: 1LY8, C-core Enzyme Commission= 0.57, TM score = 0.91). Active sites residues are: Arg70, His74, Asn111.
